# Helminth Parasites of Zoonotic Importance in Dog Faeces of North-Western Region of Pakistan: An Environmental Threat to Public Health

**Published:** 2020-05

**Authors:** Wali KHAN, Ata ULLAH, Shakeel AHMAD, Yasir INAM

**Affiliations:** Laboratory of Parasitology, Department of Zoology, University of Malakand, Lower Dir, Pakistan

## Dear Editor-in-Chief

The role of the dog as zoonotic reservoirs has received little attention from scientists and public health executives all over the world including Pakistan. This study reports a cross-sectional survey of zoonotic parasites of dogs living surrounding human settlements in district lower Dir, Pakistan. Dog feces are the main pollution source in the environment. Vehicular traffic, as well as the wind, can help in spreading pathogens present in dog feces, contaminating food which may later be a source of infection (1). “Parasite eggs can also be carried into human houses if adhered to shoes or animals’ paws” (2). Additionally, arthropods and other environmental factors, like the rain, air, and wind may also play a vital role in this context. Environment contaminated with dog feces, harboring various infective stages of parasites such as eggs, larvae or oocysts acts as a leading source of infection to live-stock and human (3). Being a reservoir host for a large number of parasites dogs share these pathogens between pets and humans (4). Human can be infected through the ingestion of eggs, cysts or oocysts via contaminated food-stuffs or water, hands, inhalation of dust, and/or by penetration of larvae through the skin (5). Geophagia and barefoot walking are the common risk factors of infection (6).

Overall, 152 faecal samples of dogs were collected from surrounding the residential areas in lower Dir, Khyber Pakhtunkhwa, Pakistan from Mar to Aug 2017. The samples were first checked with naked eye for the presence of any adult stage of the parasites and then these were prepared for microscopic examination. The results revealed 26.8% prevalence of infection overall with intestinal helminth parasites. Almost half of the dogs were found to be infected with single and the other half with mixed parasite species (Table 1). *Diphylidium caninum* was the frequently detected species of helminth followed by *Taxocara canis, Taenia* spp., *Ancylostoma caninum* while *Taxoascaris* spp., *Capilaria* spp., and *Trichuris vulpis* were the least prevalent parasite species detected (Table 2).

**Table 1:** Association of parasites: monoparasitism and polyparasitism among dogs living surrounding the residential areas of lower Dir district, Pakistan

***Type of infection***	***No.of species***	***Species associated***	***Total***
Mono-parasitism	1 species (n=27)	*Toxocara canis*	6
		*Capillaria* spp.	2
		*Ancylostoma caninum*	3
		*Dipylidium caninum*	11
		*Taenia* spp.	5
Poly-parasitism	2 species (n=13)	*T. canis+A.caninum*	3
		*T.canis+D.caninum*	4
		*T.vulpis*+*Taenia* spp.	2
		*Toxosscaris +Taenia* spp	2
		*Toxocara canis+D.caninum*	2
	3 species (n=1)	*T.canis+D.caninum+Taenia* spp.	1
Total poly-parasitism			14
Total of infected dogs			41

**Table 2:** Prevalence of parasites in faeces of dogs living surrounding the residential areas of lower Dir district, Pakistan

***Parasites***	***Total***	***Prevalence (%)***
*Dipylidium caninum*	18	11.8
*Toxocara canis*	16	10.5
*Taenia* spp.	10	6.57
*Ancylostoma caninum*	6	3.94
*Toxoascaris* spp	2	1.31
*Capillaria* spp.	2	1.31
*Trichuris vulpis*	2	1.31
Total infected	56	36.8
Total examined	152	152

A small number of reports are available on the infection risk of dogs to humans in Pakistan. These dogs have frequent contact with other animals, their faeces, and a variety of refuse and food-stuffs that contain zoonotic agents, which promotes infection with a variety of zoonotic agents and subsequent human exposure. The present study indicated that dog act as a potential public health risk, transmit infective forms of parasites to humans. Dog feces play a key role in environmental contamination than other animals. The general public and dog owners should be aware of the presence of dog parasites in their surroundings.
